# High Thermal Resistance of Epoxy/Cyanate Ester Hybrids Incorporating an Inorganic Double-Decker-Shaped Polyhedral Silsesquioxane Nanomaterial

**DOI:** 10.3390/molecules27185938

**Published:** 2022-09-13

**Authors:** Yang-Chin Kao, Wei-Cheng Chen, Ahmed F. M. EL-Mahdy, Meei-Yu Hsu, Chih-Hao Lin, Shiao-Wei Kuo

**Affiliations:** 1 Department of Materials and Optoelectronic Science, Center for Functional Polymers and Supramolecular Materials, National Sun Yat-Sen University, Kaohsiung 80424, Taiwan; 2 Material and Chemical Research Laboratories, Industrial Technology Research Institute, Chutung, Hsinchu 31040, Taiwan; 3 Department of Medicinal and Applied Chemistry, Kaohsiung Medical University, Kaohsiung 807, Taiwan

**Keywords:** epoxy, cyanate ester, POSS, thermal stability, nanocomposites

## Abstract

In this study, we prepared a difunctionalized cyanate ester double-decker silsesquioxane (DDSQ-OCN) cage with a char yield and thermal decomposition temperature (*T*_d_) which were both much higher than those of a typical bisphenol A dicyanate ester (BADCy, without the DDSQ cage) after thermal polymerization. Here, the inorganic DDSQ nanomaterial improved the thermal behavior through a nano-reinforcement effect. Blending the inorganic DDSQ-OCN cage into the epoxy resin improved its thermal and mechanical stabilities after the ring-opening polymerization of the epoxy units during thermal polymerization. The enhancement in the physical properties arose from the copolymerization of the epoxy and OCN units to form the organic/inorganic covalently bonded network structure, as well as the hydrogen bonding of the OH groups of the epoxy with the SiOSi moieties of the DDSQ units. For example, the epoxy/DDSQ-OCN = 1/1 hybrid, prepared without Cu(II)-acac as a catalyst, exhibited a glass transition temperature, thermal decomposition temperature (*T*_d_), and char yield (166 °C, 427 °C, and 51.0 wt%, respectively) that were significantly higher than those obtained when applying typical organic curing agents in the epoxy resin. The addition of Cu(II)-acac into the epoxy/BADCy and epoxy/DDSQ-OCN hybrids decreased the thermal stability (as characterized by the values of *T*_d_ and the char yields) because the crosslinking density and post-hardening also decreased during thermal polymerization; nevertheless, it accelerated the thermal polymerization to a lower curing peak temperature, which is potentially useful for real applications as epoxy molding compounds.

## 1. Introduction

Epoxy resins are the most common thermosetting polymers for high-performance applications (e.g., coatings, adhesives, printed circuit boards) in, for example, the aerospace industry and for microelectronic encapsulation, with those featuring aromatic units receiving much current interest [1,2,3,4,5,6]. In general, the high crosslinking densities that arise when using a triglycidyl ether of *p*-aminophenol or tetraglycidyl diamino diphenylmethane as the crosslinking agent result in epoxies with high glass transition temperatures (*T*_g_) but very brittle properties [7,8,9,10]. Blending thermoplastic polymers (e.g., flexible block copolymers) into epoxy resins can enhance their toughness, as a result of intermolecular interactions occurring after the ring-opening polymerization of the epoxy units forms hydroxyl (OH) groups [11,12,13,14]. Although epoxy technology has been studied for many years, typical epoxy resins are still difficult to employ in applications requiring temperatures higher than 150 °C, even when they possess high crosslinking densities of highly aromatic units in their backbones [14,15,16].

Improvements in the thermal stabilities of epoxy resins will be necessary for the formulation of reliable epoxy molding compounds (EMCs) necessitating high-temperature operation [17,18,19,20]. Copolymerization and blending with high-performance polymers can be simple approaches for the enhancement of the heat resistance or thermal stability of epoxy resins. Cyanate esters [21,22], bismaleimides [23,24], and polyimides [25] have been typical high-performance materials for copolymerization with epoxy resin. Polycyclotrimerization has been used to form cross-linked network structures with aromatic triazine rings through the thermal heating of cyanate ester (O-C≡N) compounds, resulting in excellent thermal stability. Cyanate ester resins possessing low dielectric constants and displaying low dielectronic losses for high-frequency applications have also received much interest because of their low polarities and large free volumes after thermal polymerization [26,27,28]. As a result, the copolymerization of cyanate esters with epoxy resins provides a high degree of freedom when designing copolymers from such monomers [21,22].

The thermal stabilities of poly(cyanate ester)s have been improved through the use of reactive functional units or through mixing with inorganic nanomaterials, including clays [27,29], graphene [30,31], carbon nanotubes [32,33], and polyhedral oligomeric silsesquioxanes (POSSs) [34,35,36,37,38,39,40]. Furthermore, the incorporation of inorganic POSS nanomaterials has improved the oxidation resistance and thermal stability, while decreasing the surface free energy, of epoxy/cyanate ester hybrids [34,35,36,37,38,39,40]. Such polymer/POSS hybrids are generally classified into chain-end or side-chain types, prepared from mono-functionalized or multi-functionalized POSS nanomaterials, with the latter forming insoluble cross-linked structures [41]. For example, octa-functionalized cyanate ester or epoxy POSS nanomaterials have been used previously to form epoxy/cyanate ester/POSS hybrids [34,35,36,37,38,39,40], but these multi-functionalized POSS nanomaterials have not formed well-defined crosslinked structures due to complexities arising from their three-dimensional (3D) geometries [23], with the residual cyanate ester or epoxy groups making the resulting epoxy resins unsuitable for high-performance electronics applications [22].

Recently, double-decker-shaped polyhedral silsesquioxanes (DDSQs) have been used as bifunctionalized POSS derivatives to form the main chain-type polymer/DDSQ hybrids with polyimides, phenolics, polyurethanes, polybismaleimides, and polybenzoxazines [23,42,43,44,45,46]. We have also employed a bi-functionalized cyanate-ester DDSQ nanomaterial to provide new organic/inorganic hybrids in which the O-C≡N units undergo cyclotrimerization, forming a high concentration of s-triazine rings after thermal polymerization, with a large surface area (392 m^2^/g) and mesoporous (ca. 5.6 nm) characteristics [47]. Therefore, we expected that incorporating a DDSQ-OCN derivative into the epoxy might enhance the thermal stability of the resulting epoxy resin, because of the intrinsic inorganic properties of DDSQ-OCN and its physical dispersion through noncovalent (hydrogen bonding) and covalent (the copolymerization of cyanate ester and epoxy units) bonding. In this study, we synthesized epoxy resin/DDSQ-OCN hybrids and characterized them using various thermal and spectroscopic techniques, comparing their properties with those of typical epoxy/cyanate ester hybrids prepared without DDSQ nanomaterials, including bisphenol A cyanate ester (BADCy).

## 2. Results

### 2.1. Preparation of the BADCy Monomer

**Figure 1:**
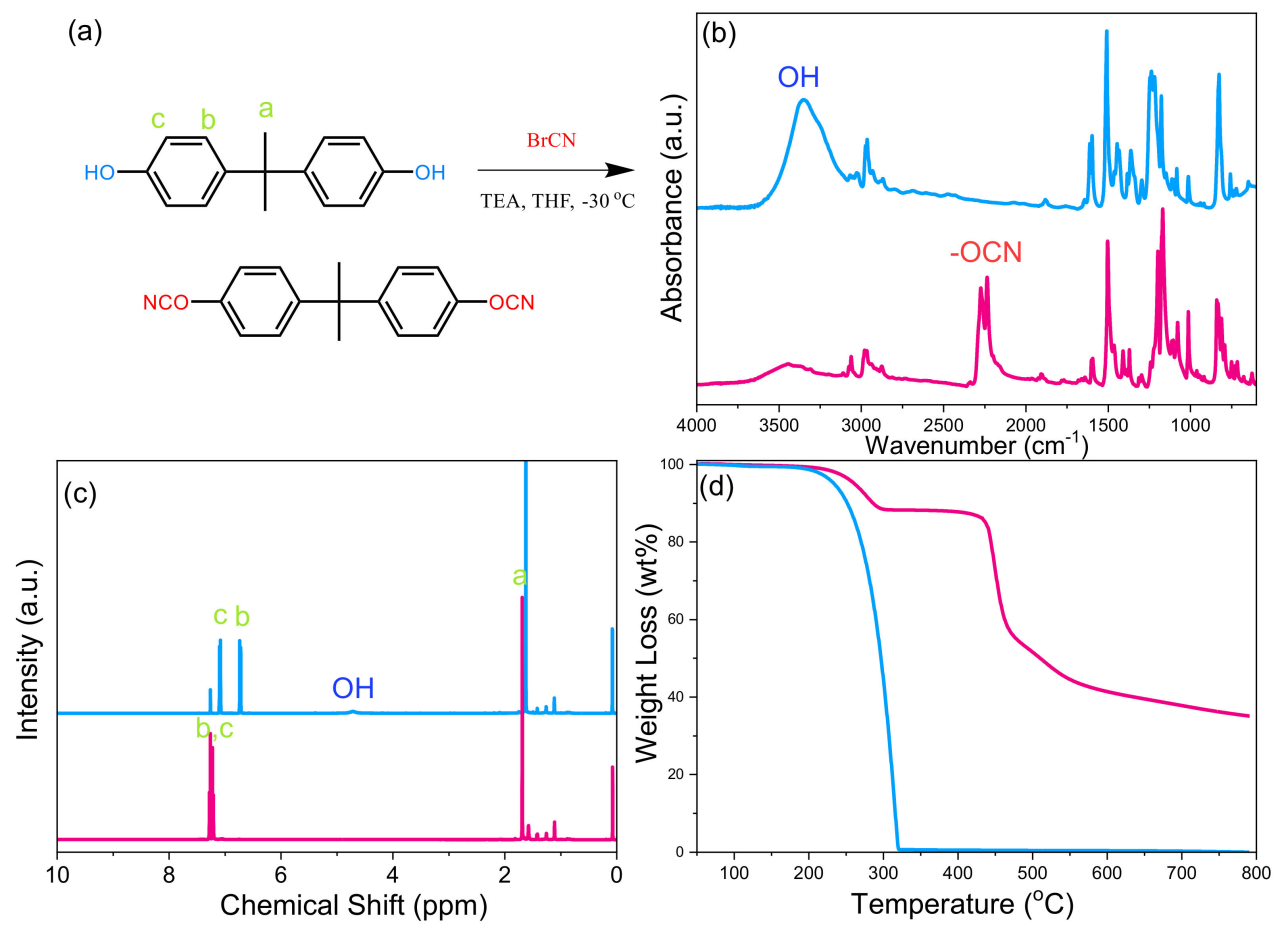
(**a**) Synthesis and (**b**) FTIR spectrum, (**c**) ^1^H NMR spectrum, and (**d**) TGA analysis of the BADCy monomer.

**Scheme 1:**
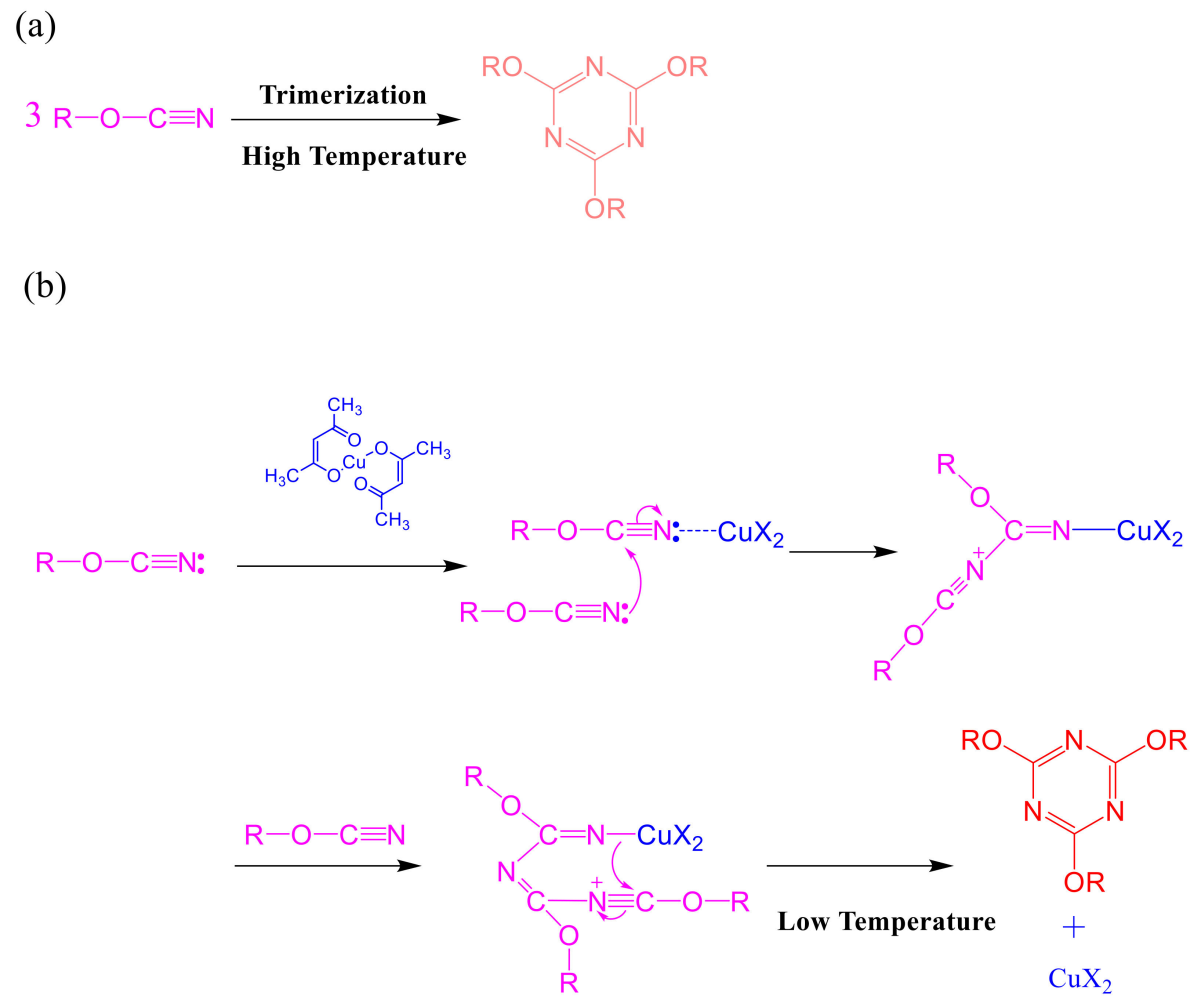
Mechanism of the cyclotrimerization of the cyanate ester resin to form the triazine rings (**a**) without a catalyst at a high temperature, and (**b**) with Cu(II)-acac as a catalyst at a low temperature.

Figure 1a displays the procedure used for the synthesis of BADCy; we confirmed its structure using Fourier transform infrared (FTIR) spectroscopy, ^1^H NMR spectroscopy, and thermogravimetric analysis (TGA) (Figure 1b–d) [47]. The FTIR spectra of bisphenol A and BADCy featured strong signals at 2235 and 2274 cm^−1^, corresponding to the O-C≡N functional groups; the disappearance of the signals for OH units in the range from 3100 to 3500 cm^−1^ indicated that the substitution reaction was complete. Figure 1c presents the ^1^H NMR spectra of bisphenol A and BADCy. The signals for the aromatic CH protons were doublets of doublets, with the electron-donating OH groups making them highly shielded, with large coupling constants. After the reaction of BPA with BrCN, the absence of a signal for the OH groups at 4.71 ppm confirmed the formation of the dicyanate esters. The aromatic CH units became more deshielded and the coupling constants of the doublets of doublets decreased because the O-C≡N units were electron withdrawing. In addition, the values obtained after the integration of the various signals (Figure S1) were consistent with the theoretical predictions. Figure 1d presents the TGA analyses of BPA and BADCy monomers. The thermal degradation temperature and char yield both increased after the substitution reaction had formed BADCy, because the O-C≡N units could react to produce the triazine structure depicted in Scheme 1a, thereby inhibiting oxygen contact and carbon residue formation [21,22].

### 2.2. Thermal Polymerization of Epoxy/BADCy Hybrids

**Figure 2:**
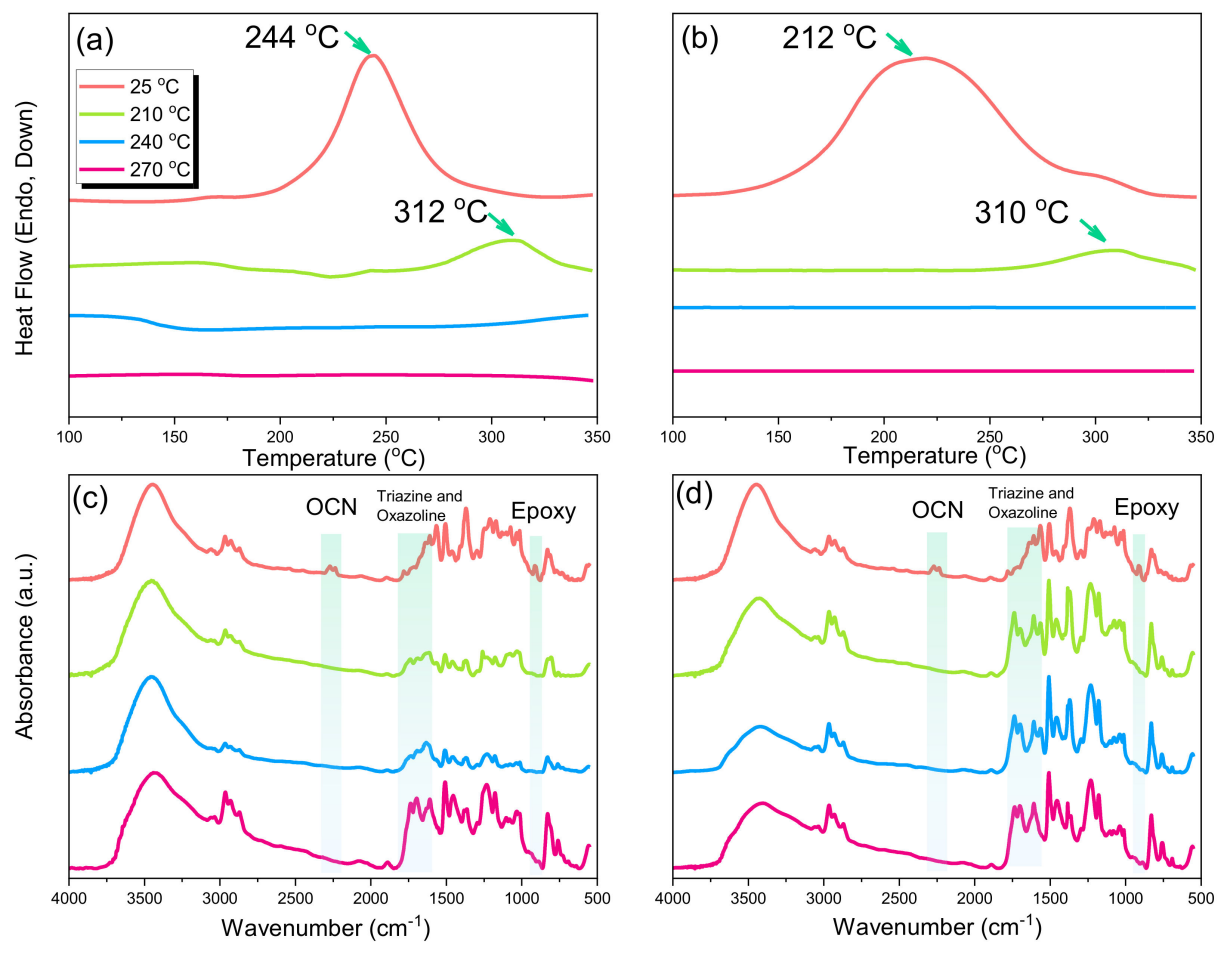
First-heating-scan DSC thermograms and FTIR spectral analyses of epoxy/BADCy hybrids measured before and after thermal polymerization at 210, 240, and 270 °C: (**a**,**c**) without a catalyst, and (**b**,**d**) with a Cu(II)-acac catalyst.

Figure 2 displays differential scanning calorimetry (DSC) traces of the epoxy/BADCy = 1/1 hybrid prepared without a catalyst (Figure 2a) and the epoxy/BADCy = 1/1 hybrids prepared with Cu(II)-acac as a catalyst (0.01 wt%) (Figure 2b), measured at a heating rate of 20 °C min^−1^. The thermal polymerization peaks for the epoxy/BADCy and epoxy/BADCy/Cu(II)-acac hybrids appeared at 244 and 212 °C, respectively. Upon increasing the thermal polymerization temperature of the epoxy resin, the thermal polymerization peaks vanished. When we performed the thermal polymerization at 210 °C, the ring-opening polymerization of the remaining epoxy groups was characterized by peaks at 312 and 310 °C, respectively. Increasing the thermal polymerization temperature to 240 and 270 °C, however, caused the thermal polymerization peaks to disappear, which was indicative of complete thermal curing. These DSC analyses revealed that the addition of Cu(II)-acac as a catalyst decreased the thermal curing temperature; Scheme 1b presents a possible mechanism for the formation of the triazine structure at a relatively lower temperature, compared with that (Scheme 1a) occurring in the absence of the Cu(II)-acac catalyst [21,22].

**Scheme 2:**
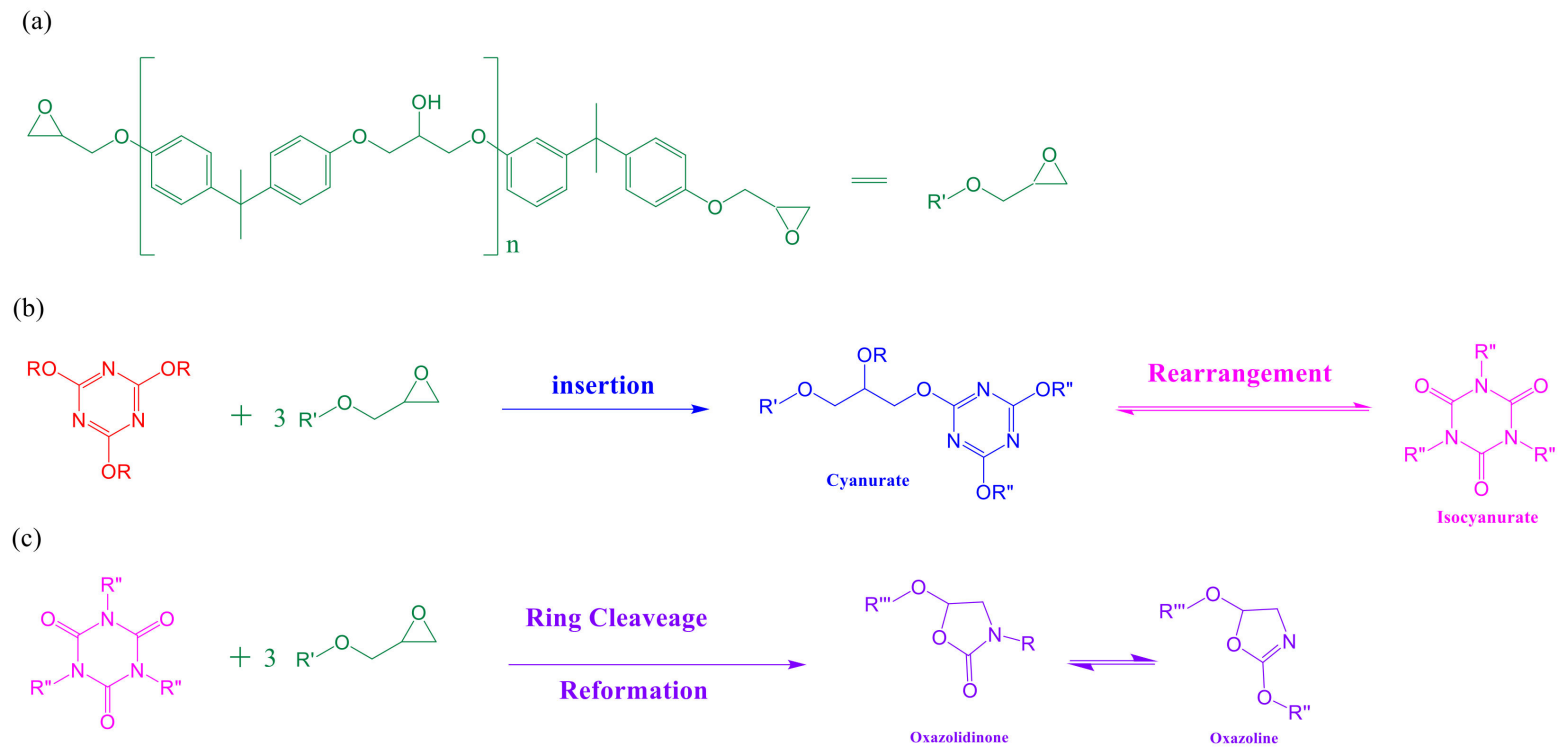
Ring-opening reactions of epoxy with triazine rings to form isocyanurateamine, oxazolidinone, and oxazoline functional groups of (**a**) the chemical structure of DGEBA type of epoxy resin, (**b**) triazine react with epoxy resin to form cyanurate and isocyannurate, and (**c**) isocyannurate react with epoxy resin to form oxazolidinone and oxazoline.

In order to examine the mechanisms of the thermal polymerizations of the epoxy/BADCy hybrids in the presence and absence of Cu(II)-acac, we recorded the FTIR spectra of these hybrids before and after thermal polymerization at 210, 240, and 270 °C [(Figure 2c,d), respectively]. The spectrum of pure BADCy featured signals for the O-C≡N units at 2235 and 2274 cm^−1^. The spectra of the pure DGEBA-type of epoxy resin exhibited absorption peaks at 914 cm^−1^ for the epoxy units in both Figure 2c,d; the signals for the O-C≡N and epoxy units both disappeared, however, after thermal polymerization at 210, 240, and 270 °C. Figure S2 reveals that pure BADCy displayed its thermal polymerization peak at 306 °C. The epoxy resin itself could catalyze the cyclotrimerization of O-C≡N units, because its addition—without and with Cu(II)-acac—decreased this thermal polymerization temperature to 244 and 212 °C, respectively. The broad absorption from 3150 to 3450 cm^−1^, representing the stretching of secondary OH groups, suggested that the ring-opening thermal polymerization of the epoxy led to isocyanurate (Scheme 2b), oxazolidinone (Scheme 2c), and triazine (Scheme 2b) units, characterized by signals at 1737, 1700, 1613, 1507, 1358, and 1227 cm^−1^ arising from the BADCy units. Scheme 2 presents a possible mechanism for the thermal polymerization of the epoxy/BADCy blend [21,22].

**Figure 3:**
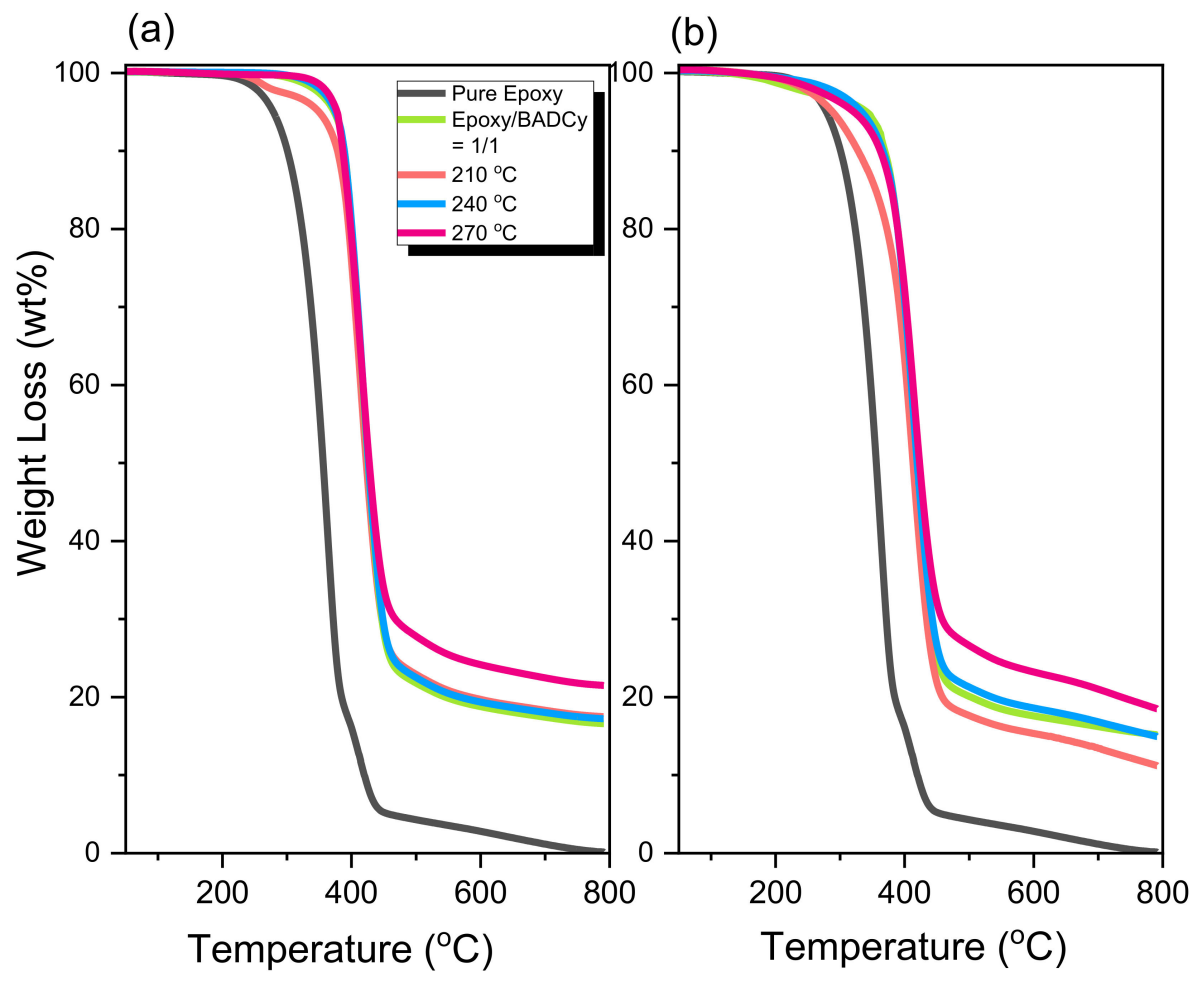
TGA traces of epoxy/BADCy hybrids in (**a**) the absence and (**b**) the presence of Cu(II)-acac, recorded before and after thermal polymerization at 210, 240, and 270 °C.

Figure 3a,b displays the TGA analyses of epoxy/BADCy hybrids (weight ratio, 1/1) in the absence and presence of Cu(II)-acac, recorded before and after thermal polymerization at various temperatures. The values of the thermal decomposition of 10 wt% (*T*_d10_) and the char yield both increased after applying the various thermal polymerization temperatures, which is consistent with the formation of crosslinked structures in the epoxy/BADCy hybrids to enhance the thermal properties. Increasing the thermal polymerization temperature led to increases in the values of *T*_d10_ and the char yield in both cases [with and without Cu(II)-acac] as a result of the O-C≡N units forming triazine structures, as displayed in Scheme 1 and Scheme 2. The presence of triazine structures minimized the decomposition of the organic material because they prevented contact with oxygen and the formation of carbon residue. For example, the values of *T*_d10_ and the char yield increased from 378 to 386 °C and from 17.5 to 21.5 wt%, respectively, for the epoxy/BADCy hybrid, and from 327 to 362 °C and from 11.2 and 18.5 wt%, respectively, for the epoxy/BADCy/Cu(II)-acac hybrid after thermal polymerization. The values of *T*_d10_ and the char yield of the epoxy/BADCy/Cu(acac)_2_ hybrid system were lower than those of the epoxy/BADCy hybrid; therefore, the catalyst decreased the thermal polymerization temperature but also decreased the crosslinking density and degree of post-hardening during the thermal polymerization procedure.

### 2.3. Thermal Polymerization of Epoxy/DDSQ-OCN Hybrids

**Figure 4:**
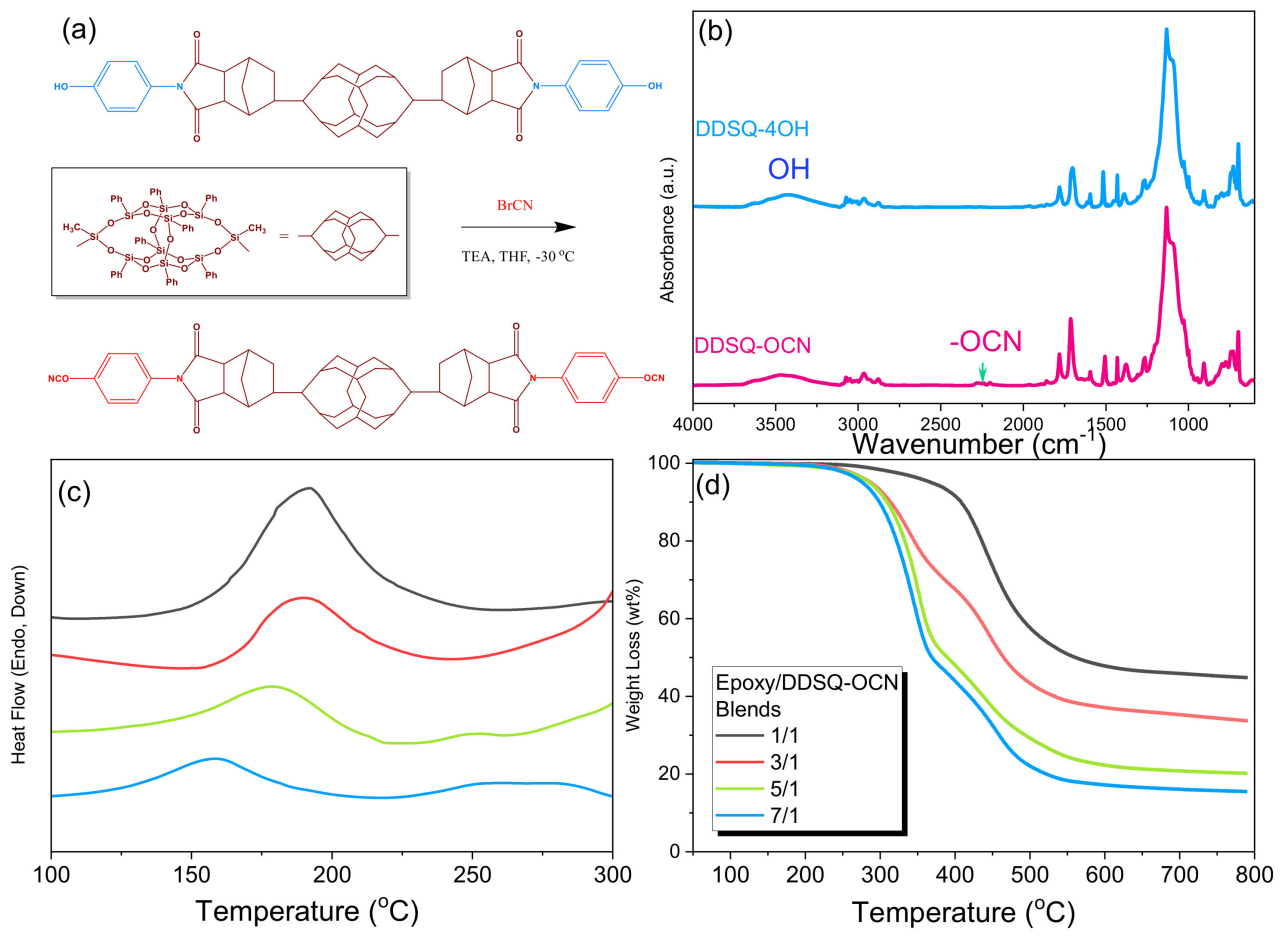
(**a**) Synthesis of the DDSQ-OCN monomer from DDSQ-4OH, and (**b**) the corresponding FTIR spectra. (**c**) First-heating-scan DSC thermograms and (**d**) TGA analyses of various epoxy/DDSQ-OCN hybrids.

Figure 4a presents the synthesis of the DDSQ-OCN monomer from DDSQ-4OH through a substitution reaction; the FTIR spectra in Figure 4b confirmed its structure, as we have discussed previously [43,47]. For example, the spectrum of the DDSQ derivative featured a weak signal for the Si–CH_3_ units at 1261 cm^−1^, and a strong signal at 1097 cm^−1^ for the Si–O–Si units; the signals for the C=O groups of DDSQ-4OH were located at 1709 and 1772 cm^−1^, with a broad signal centered at 3420 cm^−1^ representing the phenolic OH units. When the substitution reaction was complete, the spectrum of DDSQ-OCN featured signals at 2201, 2239, and 2277 cm^−1^ for the O-C≡N units, which were similar to those of the BADCy monomer in Figure 1b. Figure 4c displays the DSC thermograms of various epoxy/DDSQ-OCN/Cu(II)-acac and epoxy/DDSQ-OCN hybrids, recorded at a heating rate of 20 °C min^−1^. Upon increasing the amount of epoxy resin in the DDSQ-OCN monomer, the thermal polymerization temperature decreased, again indicating that the epoxy units themselves could catalyze the cyclotrimerization of the O-C≡N units, this time for the DDSQ-OCN monomer. For example, the thermal polymerization peak appeared at 192 °C for the epoxy/DDSQ-OCN = 1/1 hybrid; this peak shifted significantly to 158 °C for the epoxy/DDSQ-OCN = 7/1 hybrid. Furthermore, the values of *T*_d10_ and the char yield also decreased significantly, from 408 °C and 44.8 wt%, respectively, for the epoxy/DDSQ-OCN = 1/1 hybrid, and to 299 °C and 15.5 wt%, respectively, for the epoxy/DDSQ-OCN = 7/1 hybrid, with the inorganic DDSQ nanomaterial improving these thermal properties through its nano-reinforcement effect.

**Figure 5:**
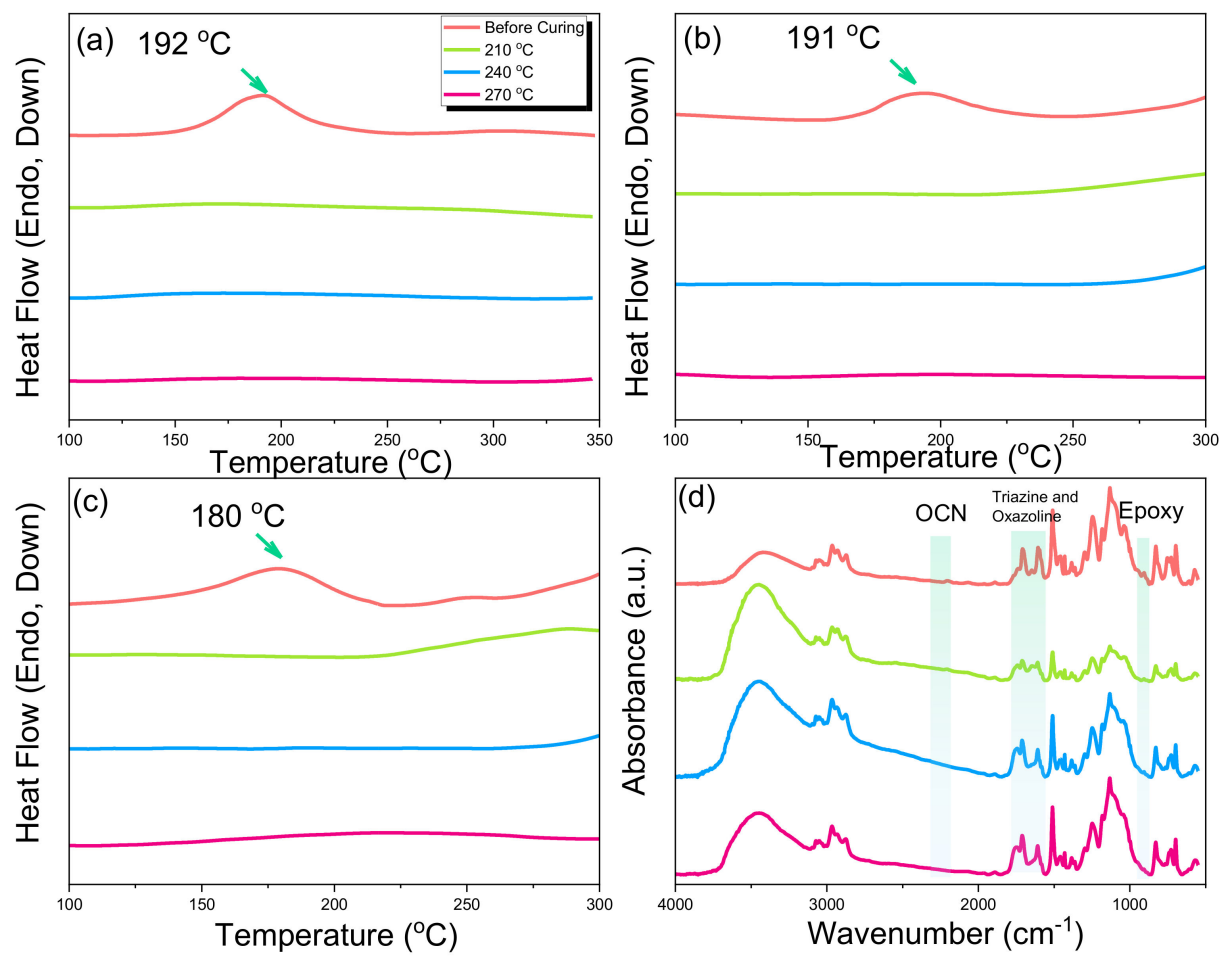
First-heating-scan DSC analyses of epoxy/DDSQ-OCN: (**a**) 1/1, (**b**) 3/1, and (**c**) 5/1 hybrids in the presence of Cu(II)-acac. (**d**) FTIR spectra of the epoxy/ DDSQ-OCN = 1/1 hybrid, recorded before and after thermal curing at 210, 240, and 270 °C.

Figure 5a–c presents DSC traces of epoxy/DDSS-OCN/Cu(II)-acac hybrids with various epoxy/DDSQ-OCN ratios, recorded before and after each thermal polymerization procedure. As mentioned above, increasing the content of epoxy resin shifted the thermal polymerization peak from 192 to 180 °C. Furthermore, the main thermal polymerization peaks at 180–192 °C vanished when the thermal polymerization temperature was 210 °C, but a broad exothermic peak was evident after thermal polymerization at 220 °C, indicating that the partially crosslinked structure inhibited the ring opening of the epoxy units and the cyclotrimerization of the O-C≡N units. Figure 5d displays the FTIR spectra of these hybrids, measured before and after thermal polymerization at 210, 240, and 270 °C. The spectrum of the epoxy resin featured an absorption peak at 914 cm^−1^, while that of DDSQ-OCN displayed signals for the O-C≡N units at 2201, 2239, and 2277 cm^−1^; both sets of signals disappeared after thermal polymerization at each temperature. Furthermore, a strong, broad signal for OH stretching appeared at 3100–3450 cm^−1^, indicating that the ring opening of the epoxy had occurred, with signals for isocyanurateamine, oxazolidinone, and triazine units appearing at 1753, 1703, 1614, 1505, 1362, and 1251 cm^−1^, arising from the O-C≡N units. In addition, the signal for the Si–O–Si units of the epoxy/DDSS-OCN hybrid at 1092 cm^−1^ shifted to 1103 cm^−1^ after thermal polymerization, suggesting that the OH units of the epoxy resin formed hydrogen bonds with the Si–O–Si units of the DDSQ cage [41]. We observed this phenomenon widely in earlier studies of hydrogen bonding in POSS nanomaterials [41,48]; here, we suspected that these secondary interactions would enhance the thermal and mechanical properties of the polymeric matrix.

### 2.4. Thermal and Mechanical Properties of Epoxy/DDSQ-OCN Hybrids

**Figure 6:**
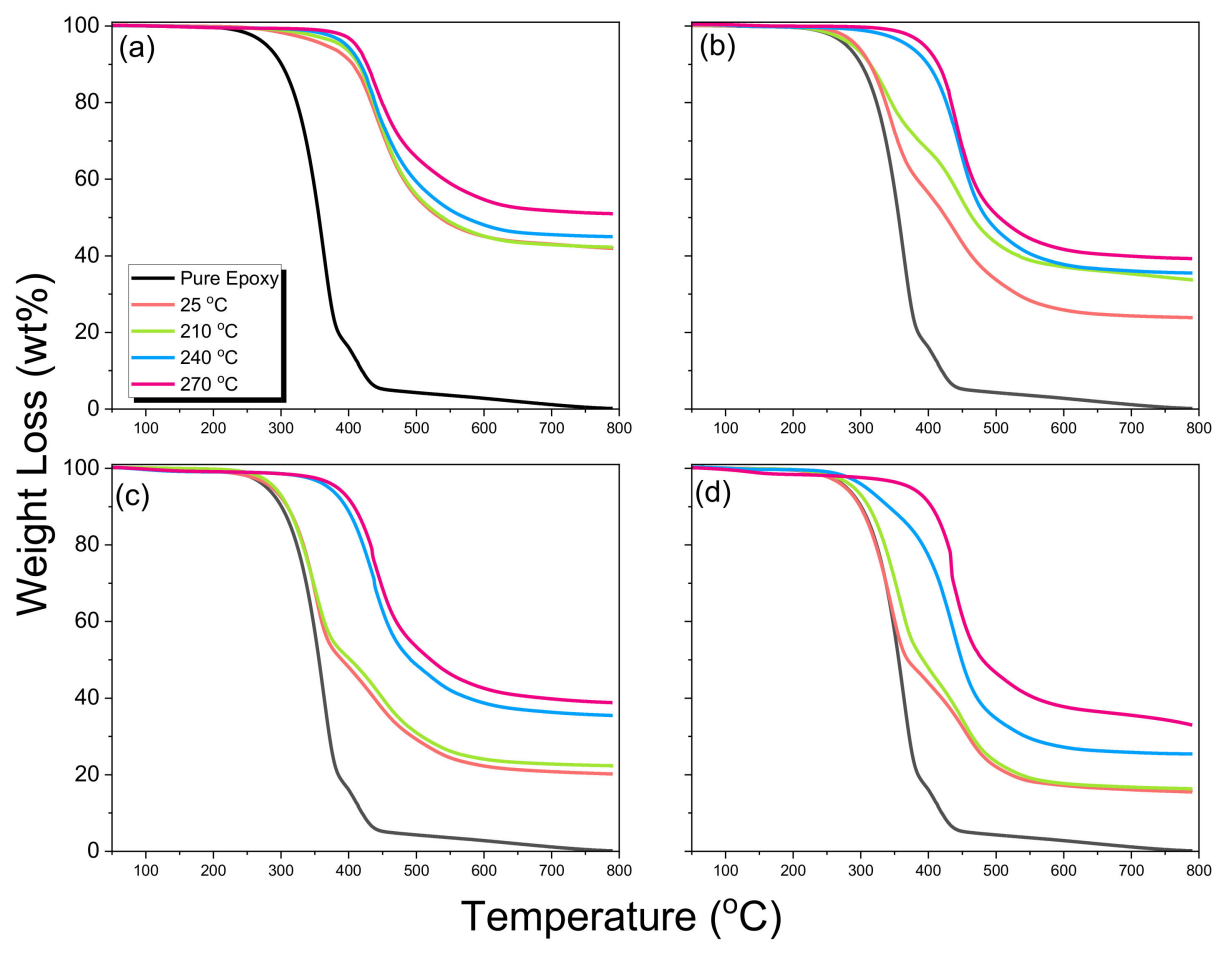
TGA traces of pure epoxy and epoxy/DDSQ-OCN: (**a**) 1/1, (**b**) 3/1, (**c**) 5/1, and (**d**) 7/1 hybrids, recorded after thermal polymerization at various temperatures.

**Figure 7:**
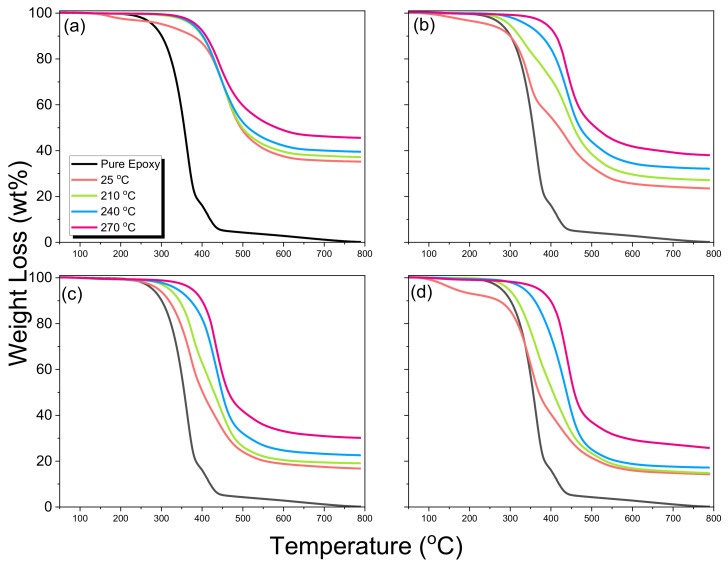
TGA traces of pure epoxy and epoxy/DDSQ-OCN: (**a**) 1/1, (**b**) 3/1, (**c**) 5/1, and (**d**) 7/1 hybrids with Cu(II)-acac, recorded after thermal polymerization at various temperatures.

**Figure 8:**
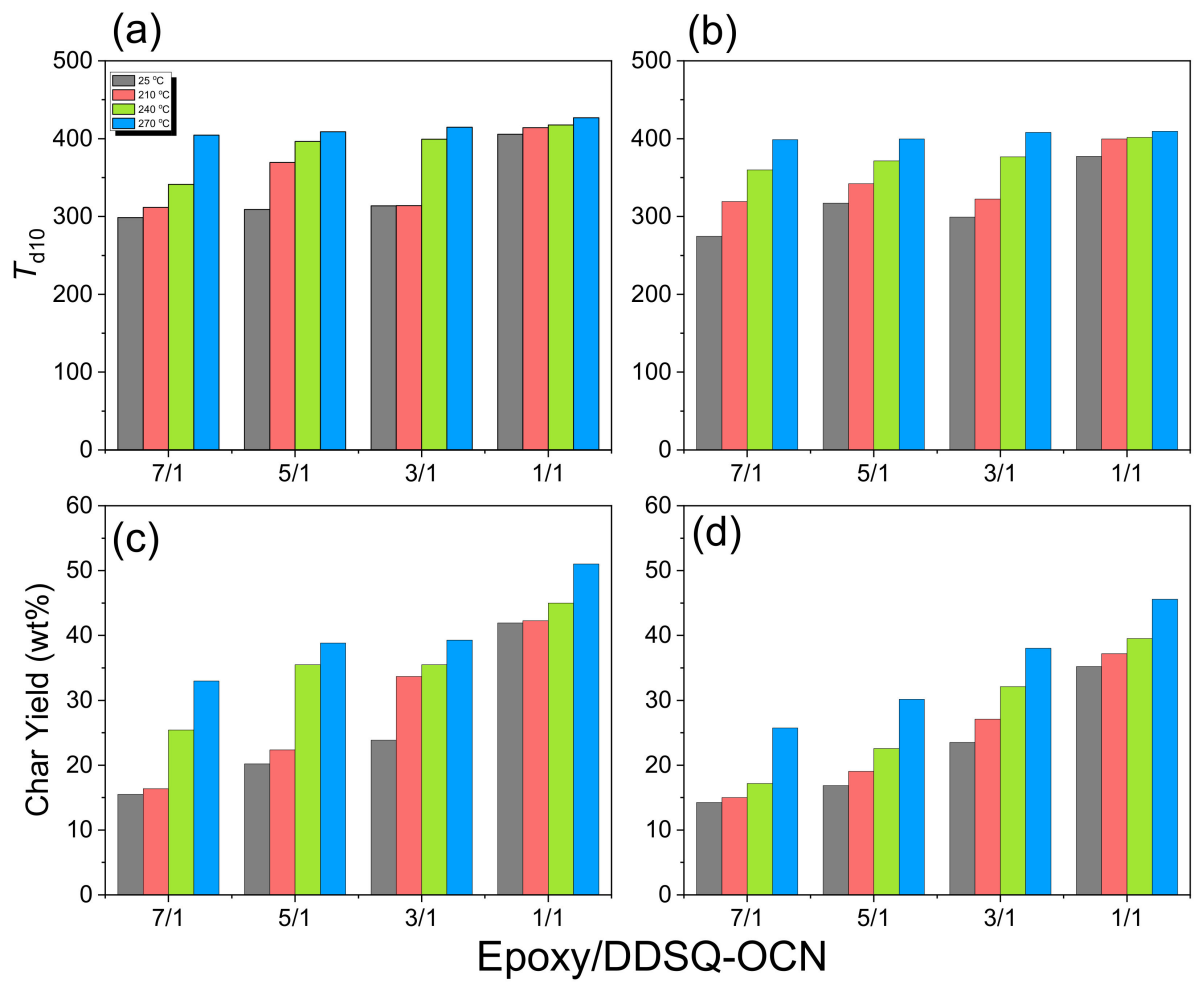
Values of *T*_d10_ and the char yields of epoxy/DDSQ-OCN hybrids in (**a**,**c**) the absence and (**b**,**d**) the presence of Cu(II)-acac, measured after thermal polymerization at various temperatures.

Figure 6 and Figure 7 present the TGA traces of various epoxy/DDSQ-OCN hybrids, in the absence and presence of Cu(II)-acac, recorded before and after thermal polymerization at each temperature, respectively. The values of *T*_d10_ and the char yield both increased after applying the various thermal polymerization temperatures, which is consistent with a crosslinking structure of DDSQ-OCN having formed in the epoxy matrix, thereby enhancing the thermal properties, similarly to the behavior of the epoxy/BADCy systems. Furthermore, the values of *T*_d10_ and the char yield of the epoxy/DDSQ-OCN/Cu(acac)_2_ hybrids were lower than those of the epoxy/DDSQ-OCN hybrid; again, this behavior is similar to that of the epoxy/BADCy systems, with decreases in the crosslinking density and the degree of post-hardening occurring during thermal polymerization. All of the values of *T*_d10_ and the char yield of the various epoxy/DDSQ-OCN hybrids, in the presence and absence of Cu(II)-acac, are summarized in Figure 8.

**Figure 9:**
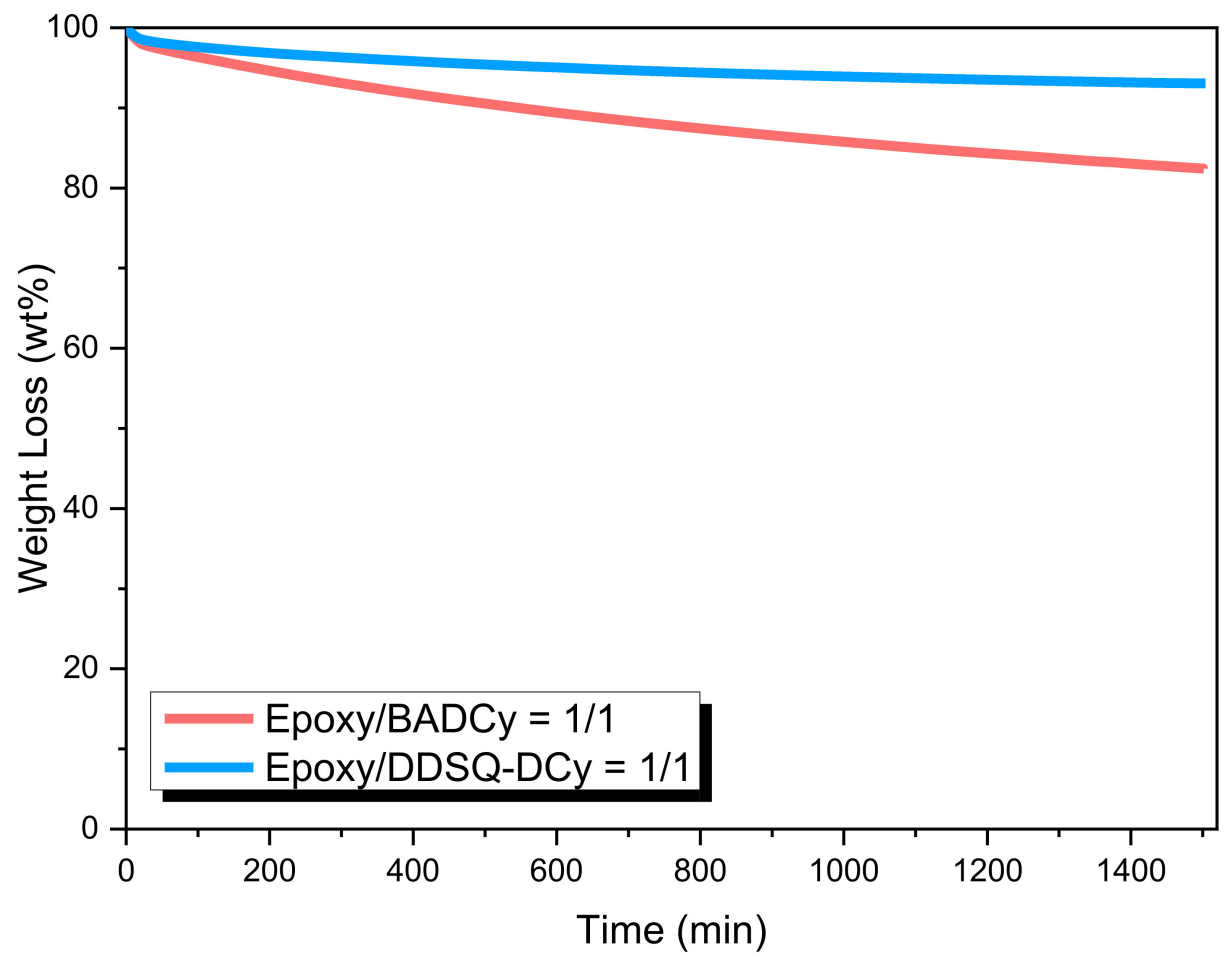
TGA profiles of epoxy/BADCy and epoxy/DDSQ-OCN = 1/1 after thermal polymerization at 270 °C and then retaining at 250 °C for 1 day.

We found that increasing the concentration of DDSQ-OCN in both the epoxy/DDSQ-OCN and epoxy/DDSQ-OCN/Cu(acac)_2_ hybrids caused the thermal stability to increase. Furthermore, the thermal stability of the epoxy/DDQQ-OCN hybrids was greater than that of the epoxy/BADCy hybrids because the inorganic DDSQ cages exerted a nano-reinforcement effect; in addition, the epoxy resin presumably formed hydrogen bonds with the DDSQ cages. For example, the values of *T*_d10_ and the char yield of epoxy/DDSQ-OCN = 1/1 were 427 °C and 51.0 wt%, respectively; these values are much higher than those (386 °C and 21.5 wt%, respectively) of epoxy/BADCy = 1/1 after thermal polymerization at 270 °C in the absence of Cu(II)-acac. Figure 9 compares the TGA traces of the epoxy/BADCy and epoxy/DDSQ-OCN hybrids after thermal polymerization at 270 °C and sitting at 250 °C for various periods of time. We conclude that the presence of the inorganic DDSQ particles in the cyanate ester region improved the thermal stability, with the char yield of the epoxy/DDSQ-OCN = 1/1 hybrid (93.1 wt%) being greater than that of the epoxy/BADCy = 1/1 hybrid (82.8 wt%) after 24 h at 250 °C.

**Figure 10:**
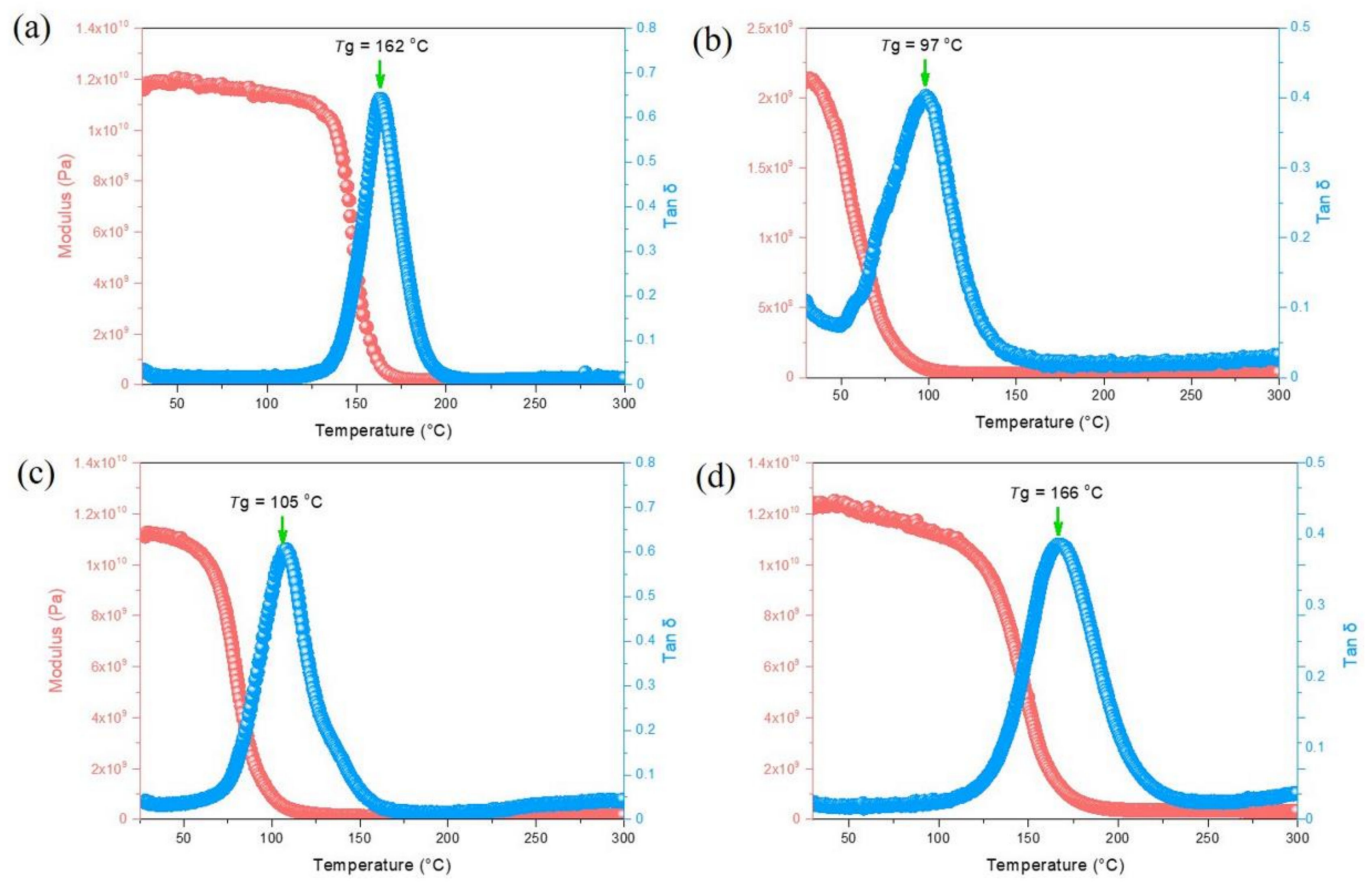
DMA thermal analyses of (**a**) epoxy/BADCy = 5/1 and (**b**) epoxy/DDSQ-OCN = 5/1, (**c**) 3/1, and (**d**) 1/1 hybrids, recorded after thermal polymerization at 270 °C.

Figure 10 displays the results of the dynamic mechanical analysis (DMA) of the epoxy/BADCy = 5/1 and various epoxy/DDSQ-OCN hybrids after thermal polymerization at 270 °C, implying their loss, tan δ, corresponding to the *T*_g_ value and storage modulus (*E*′) due to the mechanical property. The initial storage modulus of epoxy/BADCy = 5/1 at 25 °C was 11,608 MPa, with a value of *T*_g_ of 162 °C. The epoxy/DDSQ-OCN = 5/1 hybrid exhibited lower initial values of *E*′ and *T*_g_ (2093 MPa and 97 °C, respectively), presumably because the molecular weight (*M*_w_) of DDSQ-OCN (1713 g/mol) was approximately six times that of BADCy (278 g/mol), and thus the molar ratio of DDSQ-OCN relative to epoxy was lower than that of BADCy at the same weight ratio. As a result, the poorer mechanical properties and lower value of *T*_g_ arose because of a lower crosslinking density in the epoxy/DDSQ-OCN hybrid at the same weight ratio, compared with that in the epoxy/BADCy hybrid. Further increasing the concentration of DDSQ-OCN in the epoxy resin caused the values of *E*′ and *T*_g_ to increase to 11,110 MPa and 105 °C, respectively, for epoxy/DDSQ-OCN = 3/1, and to 12,410 MPa and 166 °C, respectively, for epoxy/DDSQ-OCN = 1/1, due to the rigid inorganic DDSQ nanoparticles enhancing the nano-reinforcement effect, while also increasing the crosslinking of the structure and decreasing the mobility of the epoxy resin. Comparing the epoxy/BADCy= 5/1 and epoxy/DDSQ-OCN = 1/1 hybrids with similar molar ratios of their cyanate ester functional units, the presence of DDSQ-OCN appeared to provide superior mechanical properties and values of *T*_g_ when compared with the effects of BADCy; thus, incorporating the cubic DDSQ cages could indeed improve the thermal and mechanical properties.

**Figure 11:**
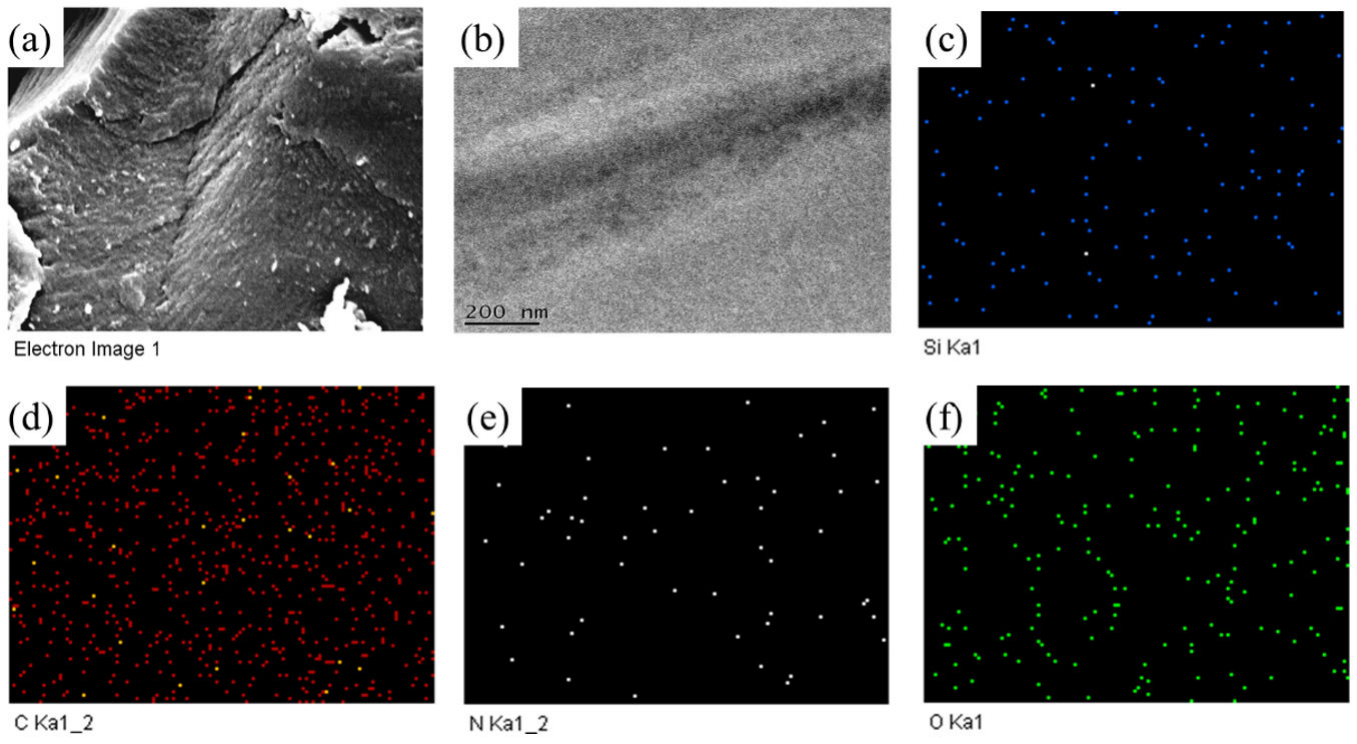
(**a**) SEM and (**b**) TEM images of the epoxy/DDSQ-OCN = 1/1 hybrid. (**c**) Si, (**d**) C, (**e**) N, and (**f**) O mapping of the SEM images after thermal polymerization.

Scanning electron microscopy (SEM) and transmission electron microscopy (TEM) revealed the dispersion of inorganic DDSQ-OCN cages in the epoxy resin. Figure 11a,b display SEM and TEM images, respectively, of the epoxy/DDSQ-OCN = 1/1 hybrid after thermal polymerization at 270 °C. Both images reveal a featureless morphology without macro-phase separation, indicating that the inorganic DDSQ nanoparticles were dispersed uniformly in the epoxy matrix. Furthermore, Si and C mapping, as well as N and O mapping, based on SEM analyses, also revealed the homogeneous dispersion of DDSQ cages on the surface of the epoxy resin. Moreover, the blue points for the Si mapping in Figure 11c indicate DDSQ-rich domains, again suggesting highly dispersed cubic DDSQ cages in the epoxy matrix that inhibited chain mobility, thereby improving the thermal properties. This result is consistent with our TGA and DMA thermal analyses. Thus, the inorganic nanoparticles themselves had an effect, as did the intermolecular hydrogen bonding between the epoxy and DDSQ cage structures after thermal polymerization, as revealed through FTIR spectral analyses.

## 3. Experimental Section

### 3.1. Materials

The bisphenol A, tetrahydrofuran (THF), and Cu(II) 2,4-pentanedionate (Cu(II)-acac) were purchased from Alfa–Aesar. The triethylamine, cyanogen bromide (BrCN), methanol (MeOH), and cyclohexane were purchased from Sigma–Aldrich. The DDSQ-OCN was synthesized as described previously [47]. The epoxy resin (DGEBA, DER 331) was purchased from Dow Chemical (Midland, MI, USA), with an EEW of 190 g/eq.

### 3.2. Bisphenol A Cyanate Ester (BADCy)

Bisphenol A (5.00 g, 21.9 mmol) and BrCN (4.86 g, 46.0 mmol) were placed in a flask under a blanket of N_2_. THF (100 mL) was added slowly while stirring rapidly. The solution was cooled to −25 °C, and then triethylamine (3.41 mL) was added slowly over 30 min. The temperature was stabilized at −30 °C by immersion in a Dewar flask containing a MeOH/liquid N_2_ mixture. The reaction was complete after 4 h. The white salt was filtered off. Ice water (500 mL) was added to the filtrate to form a white precipitate. This crude product was recrystallized from cyclohexane to obtain a white powder (3.84 g; yield: 63%).

### 3.3. Epoxy/BADCy and Epoxy/DDSQ-OCN Hybrids

Various epoxy/BADCy and epoxy/DDSQ-OCN hybrids, in the presence and absence Cu(II)-acac (0.01 wt%), were stirred for 48 h at 60 °C under a vacuum. Each casting sample was placed into an aluminum tray and subjected to thermal polymerization at 210, 240, or 270 °C for 2 h. The epoxy hybrids were obtained with a dark brown color.

## 4. Conclusions

We prepared BADCy and DDSQ-OCN cyanate monomers through the substitution of the phenolic functional groups of BPA and DDSQ-4OH with BrCN. The char yield and value of *T*_d_ of the DDSQ-OCN monomer were higher than those of the typical BADCy monomer (without DDSQ cages) after thermal polymerization, because the inorganic DDSQ cages enhanced the thermal behavior through a nano-reinforcement effect. SEM and TEM images revealed that the inorganic DDSQ-OCN cages were dispersed homogeneously in the resulting epoxy resins. Therefore, the values of *T*_g_ and *T*_d_ and the storage modulus of these epoxy/DDSQ hybrids all increased significantly as a result of restricted chain mobility, which arose from hydrogen bonding between the OH units of the epoxy resin (after thermal polymerization) and the SiOSi units of the DDSQ cages (based on FTIR spectroscopy), as well as covalent bonding after the copolymerization of the epoxy and cyanate ester units. The values of *T*_g_ and *T*_d_ for the epoxy/DDSQ-OCN = 1/1 hybrid, prepared without Cu(II)-acac as a catalyst (166 and 427 °C, respectively, based on DMA and TGA) were significantly higher than those obtained when adding typical organic curing agents to DGEBA-type epoxy resin, because of the effect of the rigid inorganic DDSQ cage nanomaterials.

## Data Availability

The data presented in this study are available in the article and supplementary material.

## Supplementary Materials

The following supporting information can be downloaded at https://www.mdpi.com/article/10.3390/molecules27185938/s1. Figure S1: ^1^H NMR analysis of BPA and BADCy monomers. Figure S2: DSC analysis of the pure BADCy monomer.

## Sample Availability

Samples of the compounds are not available from the authors.
