# Supplementary material for: High Thermal Resistance of Epoxy/Cyanate Ester Hybrids Incorporating an Inorganic Double-Decker-Shaped Polyhedral Silsesquioxane Nanomaterial
Source: Molecules. 2022 Sep 13;27(18):5938. doi: 10.3390/molecules27185938 (PMC9502839; doi:10.3390/molecules27185938)
Supplement: Supplementary file 1 [file molecules-27-05938-s001.zip › molecules-1899275-supplementary.pdf]

Supporting Information

High Thermal Resistance of Epoxy/Cyanate Ester Hybrids Incorporating an Inorganic Double-Decker-Shaped Polyhedral Silsesquioxane Nanomaterial

Characterization

^{1}H spectra were recorded on a INOVA 500 MHz NMR spectrometer, with $CDCl_3$-*d* as an external standard. The IR spectra were measured with a Bruker Tensor 27 FTIR spectrophotometer using the conventional crystal KBr disk method. 32 scans were collected at a spectral resolution of 4 cm^{-1}. Dynamic mechanical behavior of cured sample was studied using a Du-Pont 2980 dynamic mechanical analyzer. Cured sample was polished to ≈ 3.0 × 13.0 × 30.0 mm and mounted on a single cantilever clamp. The mechanical properties were measured under nitrogen in step mode every 5 °C from 25 to 350 °C at frequency of 1 Hz. The thermal stability of the samples was characterized by using a TA Q-50 Thermogravimetric Analyzer operating under a nitrogen atmosphere. The cured sample ca. 7 mg was placed in a Pt cell and heated at a rate of 20 °C /min from 30 to 800 °C at a nitrogen flow rate of 60 mL/min. The dynamic curing kinetics was studied using a TA Q-20 instrument Differential Scanning Calorimeter operating under a nitrogen atmosphere. The sample (ca. 7 mg) was placed in a sealed aluminum sample pan. Dynamic curing scan were conducted from 30 °C to 350 °C at a heating rate of 20 °C /min.

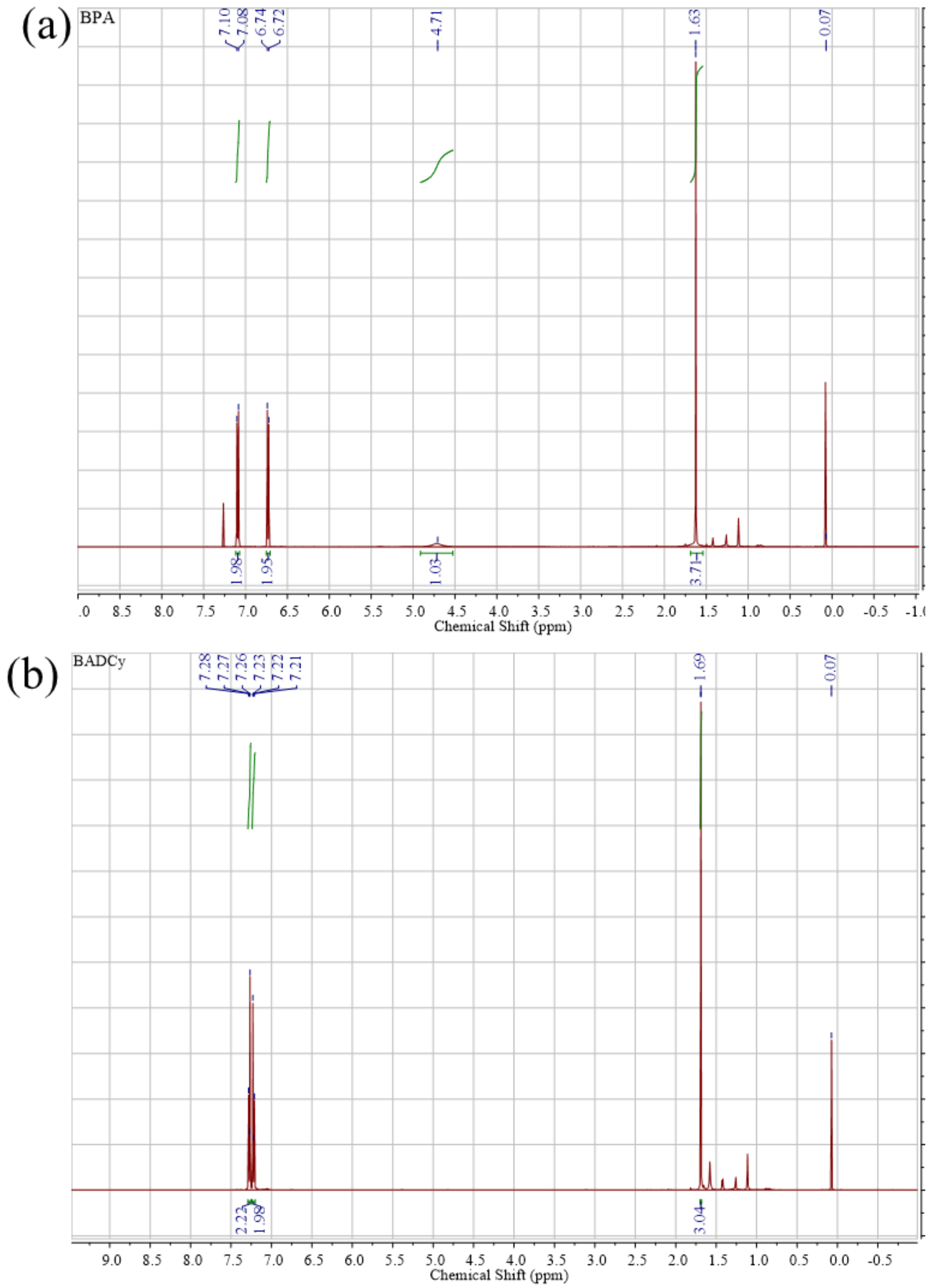

Figure S1. ^{1}H NMR spectra of (a) BPA and (b) BADCy monomer.

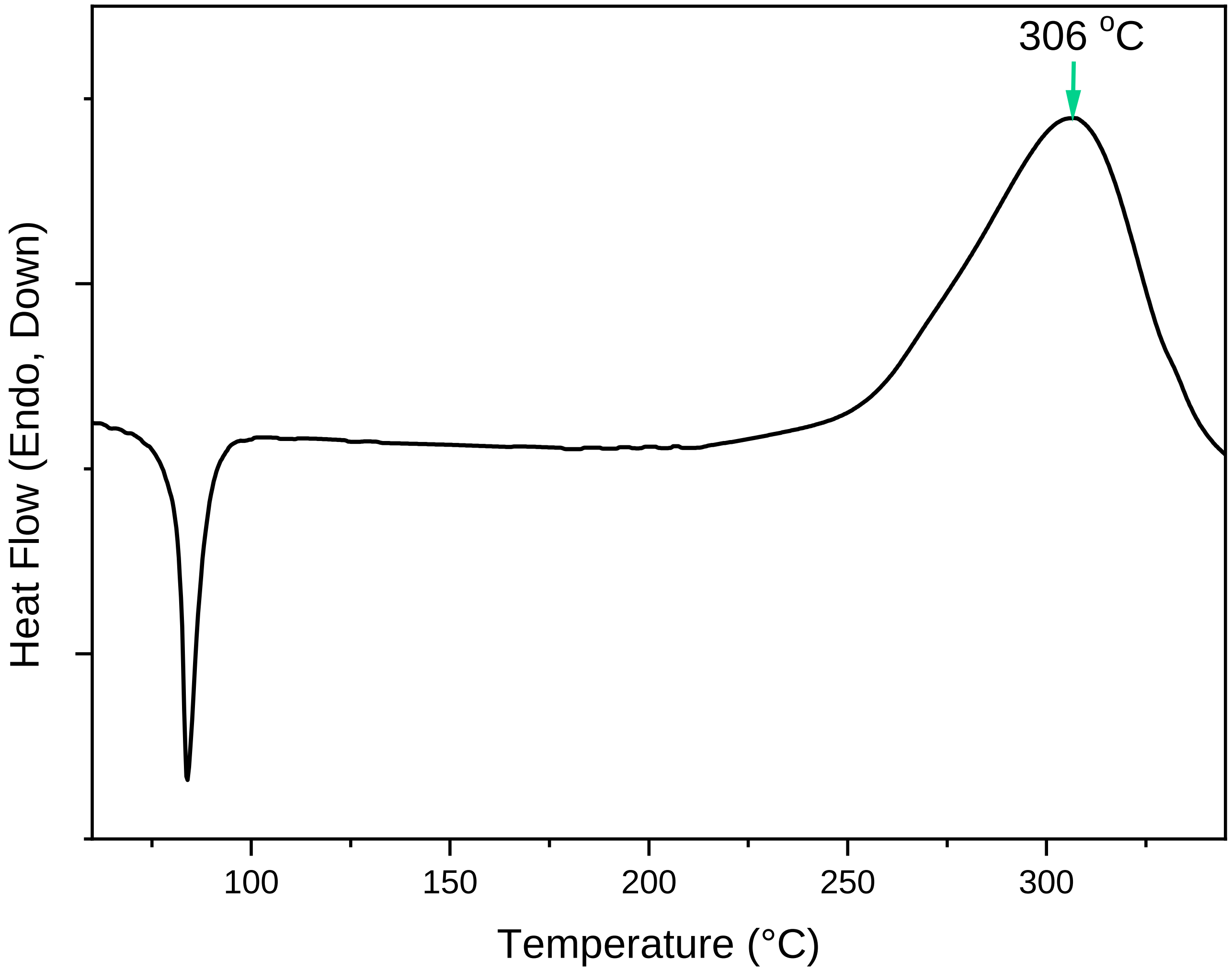

Figure S2. DSC thermal analysis of pure BADCy under first heating scan .
